# Multi-Organ Lesions in Suckling Mice Infected with SARS-Associated Mammalian Reovirus Linked with Apoptosis Induced by Viral Proteins μ1 and σ1

**DOI:** 10.1371/journal.pone.0092678

**Published:** 2014-03-24

**Authors:** Lihua Song, Yongfeng Lu, Jun He, Yonghui Yu, Tingting Zuo, Yanwei Li, Hong Zhu, Qing Duan

**Affiliations:** State Key Laboratory of Pathogen and Biosecurity, Beijing Institute of Microbiology and Epidemiology, Beijing, China; The Pirbright Institute, United Kingdom

## Abstract

We reported the isolation and characterization of a novel mammalian reassortant reovirus BYD1 that may have played an accomplice role with SARS-coronavirus during the 2003 SARS pandemic. The pathogenic mechanism of this novel reovirus is unknown. Reovirus pathogenicity has been associated with virus-induced apoptosis in cultured cells and *in vivo*. The reovirus outer capsid protein μ1 is recognized as the primary determinant of reovirus-induced apoptosis. Here, we investigated the apoptosis induced by BYD1, its outer capsid protein μ1, and its cell-attachment protein σ1 to understand the pathogenesis of BYD1. We also investigated BYD1 caused systemic complications in suckling mice. Under electron microscopy, BYD1-infected cells showed characteristics typical of apoptosis. Notably, ectopically expressed μ1 and σ1 induced similar pathological apoptosis, independent of BYD1 infection, in host cells in which they were expressed, which suggests that μ1 and σ1 are both apoptotic virulence factors. Consistent with previous reports of reovirus pathogenicity, suckling mice intracranially inoculated with BYD1 developed central nerve damage, myocarditis, and pneumonia. Collectively, our data suggest that BYD1 μ1- and σ1-induced apoptosis is involved in the multi-organ lesions in a suckling mouse BYD1 infection model.

## Introduction

Mammalian reoviruses (MRVs) are prototypical members of the family *Reoviridae*, which contains segmented double-stranded RNA (dsRNA) viruses of both medical (rotavirus) and economic (bluetongue virus) importance (reviewed in [1]–[3]). These viruses have a segmented dsRNA genome encoding eight structural proteins and three nonstructural proteins [4]. Reoviruses were originally called “*r*espiratory *e*nteric *o*rphan” viruses based on their repeated isolation from the respiratory and enteric tracts of children with asymptomatic illnesses [5]. However, there have been reports describing reoviruses associated with meningitis in infants and children [6]–[8], and with acute respiratory disease in adults [9]. Despite limited reports of severe reovirus infections in humans, pulmonary infection of mice with reovirus serotype 1 strain Lang is a very clinically relevant model of infection-induced acute viral pneumonia leading to acute respiratory distress syndrome, the most severe form of acute lung injury [10]–[13]. In a mouse infection model, reovirus-induced apoptosis is a major determinant of virulence, causing neural and cardiac injury (reviewed in [14]), and mice treated with inhibitors of apoptosis before infection have reduced tissue damage in the central nervous system and heart [15], [16]. Recent studies have indicated that the reovirus outer capsid protein μ1 is the primary factor involved in reovirus-induced apoptosis [17], [18].

We previously reported the isolation and partial characterization of a new reovirus strain, designated BYD1, isolated from the throat swabs of one patient in Beijing with severe acute respiratory syndrome (SARS) in Hep-2 cell cultures. SARS-coronavirus (SARS-CoV) was also isolated from the same samples in Vero-E6 cell cultures [19]. Three other reovirus strains, designated BLD, JP, and BYL, were isolated from other SARS patients [20]. We propose that these new reovirus strains be designated “SARS-associated mammalian reovirus” (SARS-MRV). All four SARS-MRV strains were purified by plaque assay and identified as novel members of serotype 2 by sequence analysis of their S1 segments [21]. Genome comparisons showed that BYD1 is a reassortant virus, with its S1 gene segment derived from a previously unidentified serotype 2 isolate and its other nine segments derived from ancestors of homologous serotype 1 and serotype 3 segments [21]. Notably, these SARS-MRVs have been attributed an important role in the etiology of SARS, based on the initial finding of high anti-BYD1 antibody titers in some SARS patients [19] and experimental infections showing that BYD1 can cause SARS-like symptoms in macaques and guinea pigs [22], [23].

The purpose of this study was to determine whether SARS-MRV or its proteins can induce apoptosis and to characterize the pathology of the viral infection in a murine model. To this end, we found that Hep-2 cells infected with BYD1 undergo different stages of apoptosis, characterized by nuclear and cytoplasmic shrinkage, together with various forms of chromatin margination and condensation, and followed by karyorrhexis and the formation of apoptotic bodies. We show that the ectopically expressed cell-attachment protein σ1 and the outer capsid protein μ1 can independently induce infection-like pathological apoptosis in 293 T cells. We also show that suckling mice intracranially inoculated with BYD1 display signs of central nerve damage, myocarditis, and pneumonia. The potential of viral proteins σ1 and μ1 to induce apoptosis could be associated with multi-organ injuries *in vivo*. The findings described here provide new insight into the pathogenesis of SARS-MRV.

## Materials and Methods

### Ethics statement

*The experiments described in this manuscript did not involve human subjects or nonhuman primates, so no institutional review board approval was required. The use of mice in the study was approved by the Animal Care and Use Committee at the Beijing Institute of Microbiology and Epidemiology*.

### Viruses and cell culture

SARS-MRV strain BYD1 was isolated from the throat swabs of one SARS patient, plaque purified, and confirmed of no SARS-CoV contaminants by PCR [19], [24]. Hep-2 [19] and 293 T cells were grown at 37°C under 5% CO_2_ in Dulbecco's modified Eagle's medium (Mediatech, Inc., USA) supplemented with 10% fetal bovine serum. Hep-2 cell monolayers were used for BYD1 amplification.

### Vector construction and transfection

Genomic dsRNA was purified from BYD1-infected cells as previously described [21]. The viral M2 gene was amplified using primers mu1f (5′-TCCAAGCTTATGGGGAACGCTTCCTCTATC-3′) and mulr (5′-TATGGGCCCTCAACGTGTGTACCCACGT-3′), introducing *Hin*dIII and *Apa*I sites, respectively, a SuperScript II Reverse Transcriptase kit (Life Technologies), and a PrimeSTAR HS DNA Polymerase kit (Takara). The pEGFP-C3 plasmid (Clontech) and the amplicon were then doubly digested with *Hin*dIII and *Apa*I, and the fragments were purified with a QIAEX II Gel Extraction Kit (Qiagen) and ligated using T4 DNA ligase (NE Biolabs), according to manufacturer's specifications. The ligation mixture was then transformed into chemically competent *Escherichia coli* DH5α cells. A kanamycin-resistant clone was isolated and the pEGFP-C–μ1 plasmid was purified and sequenced, confirming the plasmid to be as predicted. The vector pEGFP-C–σ1 was constructed in a similar manner using primers sigma1f (5′-TTCAAGCTTATGTCTGAGCTGATTCAGC-3′) and sigma1r (5′-AAAGGGCCCTCAGCCTAAGCATGGATACAT-3′). The plasmids were purified with an endotoxin-free plasmid purification kit (Qiagen). 293 T cells were transfected with pEGFP-C3, pEGFP-C–μ1, or pEGFP-C–σ1 using Lipofectamine 2000 (Life Technologies), according to the manufacturer's instructions.

### Microscopy

Phase-contrast images and fluorescent images of the transfected 293 T cells were captured at a magnification of ×200 using an Olympus inverted microscope and a Canon digital camera. Hep-2 cells infected with BYD1 and mock-transfected controls, and 293 T cells transfected with different vectors were fixed with 2% glutaraldehyde–0.5% paraformaldehyde in 0.1 M cacodylate buffer (pH 7.2) for subsequent standard processing, infiltration, embedding, ultrathin section preparation, and staining for high-contrast transmission electron microscopy (TEM). Electron microscopic images were taken with a Philips Tecnai-10 electron microscope.

### Flow cytometry

The apoptosis of 293 T cells was quantified by the expression of annexin V using an Annexin-V FITC Apoptosis Detection Kit (Beijing Biosea Biotechnology). Briefly, cells at 18 h posttransfection were washed once with PBS, trypsinized, washed twice with PBS, and quantified. The cells (1×10^6^) were resuspended in 200 μl of binding buffer and treated with 10 μl of fluorescein isothiocyanate (FITC)–annexin V and 5 μl of propidium iodide for 15 min at room temperature in the dark. The samples were then added to 300 μl of binding buffer and analyzed by flow cytometry (excitation 488 nm, emission 530 nm). A two-tailed Student's *t* test was used for the statistical analysis.

### Suckling mice infection experiments

Two-day-old specific-pathogen-free Kunming mice, maintained together with the mother mice under specific-pathogen-free conditions, were purchased from the Animal Center of the Academy of Military Medical Sciences of China. Ten suckling mice were assigned to two groups, each containing five mice. The infection group was inoculated intracranially with 2×10^6^ TCID_50_ of BYD1 in 20 μl of PBS. The mock infection group was inoculated intracranially with 20 μl of PBS. All the treated mice were observed daily, euthanized on the eighth day postinoculation, and their brains, hearts, lungs, and kidneys excised, fixed in 10% formalin, and embedded in paraffin blocks. The tissue sections were stained with hematoxylin–eosin (H&E) for blind pathological analysis by a veterinary pathologist.

## Results

### Apoptosis of human laryngeal cancer cells caused by SARS-MRV infection

**Figure 1:**
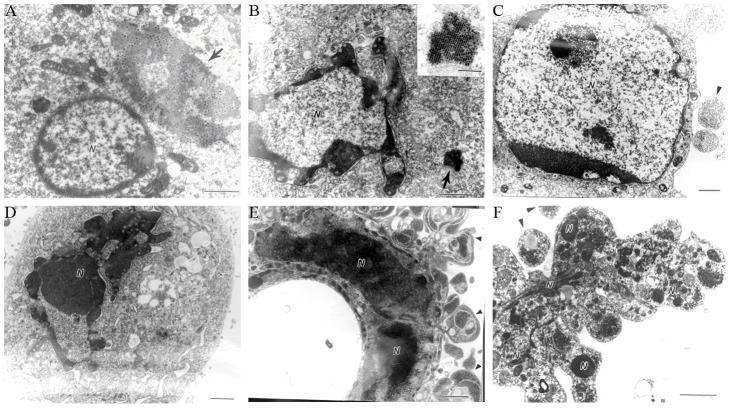
Hep-2 cells infected with SARS-MRV showed the typical characteristics of apoptosis under electron microscopy. Bar  = 1 μm. Arrowheads indicate viral inclusion bodies. (A) A viral inclusion body (arrow) near a nucleus with initial chromatin margination and condensation. (B) Typical chromatin margination and condensation in a nucleus with a remnant viral inclusion body nearby (arrow). Insert shows the remnant viral inclusion body at higher magnification (Bar  = 0.25 μm). (C) A nucleus with chromatin condensation and nearby apoptotic bodies. (D) A shrunken pyknotic nucleus. (E) A pyknotic, deformed nucleus surrounded by apoptotic bodies. (F) Shrinkage, budding, and karyorrhexis of an infected cell with apoptotic bodies.

The human laryngeal cancer cell line (Hep-2) was used to isolate all SARS-MRV strains from swabs or lung tissues from SARS patients [19], [21]. During viral passages, the Hep-2 cells infected with these viruses consistently showed typical cytopathic effects, including cell rounding and detachment. We characterized the ultrastructural cytopathology of Hep-2 cells infected with SARS-MRV strain BYD1. Cells undergoing various stages of apoptosis were easily observed with electron microscopy (Figure 1), although most apoptotic cells lacked observable viral particles or inclusion bodies in their cytoplasm. These apoptotic cells were characterized by initial cytoplasmic and nuclear shrinkage, with various degrees of chromatin margination and condensation, followed by karyorrhexis and the formation of apoptotic bodies. This finding that BYD1 infection triggers cell apoptosis is consistent with the established fact that reoviruses induce apoptosis *in vitro*.

### Ectopic expression of SARS-MRV proteins μ1 and σ1 induces pathological apoptosis

**Figure 2:**
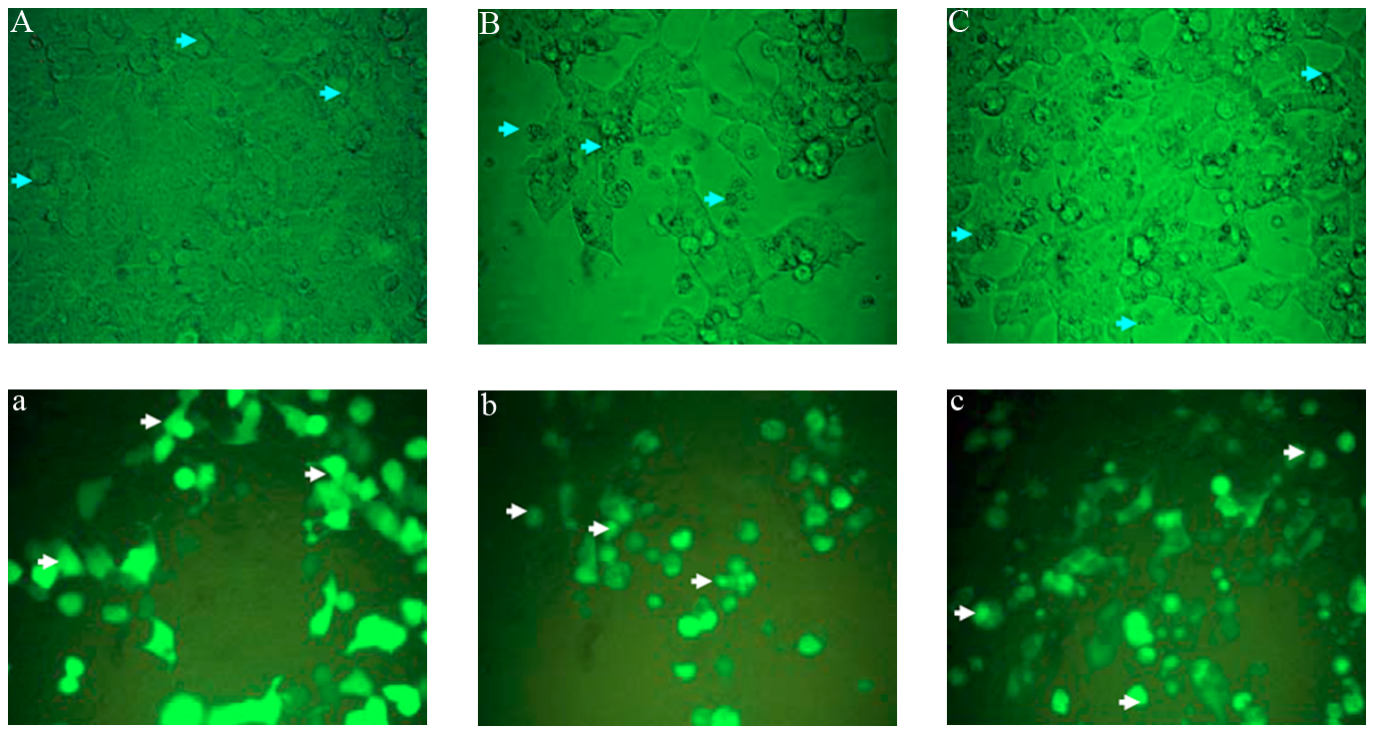
Ectopically expressed SARS-MRV μ1 and σ1 caused pathological changes in 293 T cells. Phase-contrast microscopic images of transfected cells under visible light (A–C) and ultraviolet light (a–c). Cells expressing GFP fusion proteins of μ1 (B, b) or σ1 (C, c) show cytopathic effects of cell rounding and shrinkage, compared with the healthy cell monolayer expressing GFP alone (A, a).

**Figure 3:**
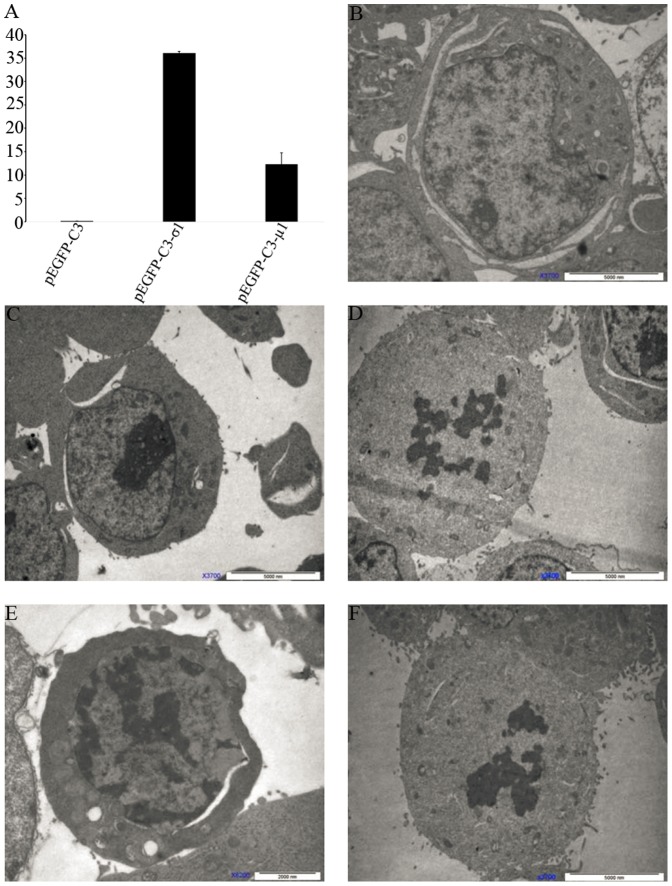
Ectopically expressed SARS-MRV μ1 and σ1 induced typical apoptosis in 293 T cells. (A) Cells transfected with pEGFP-C3–σ1 or pEGFP-C3–μ1 had a significantly higher rate of apoptosis than cells transfected with the pEGFP-C3 control vector (*p*<0.01), detected with flow cytometry at 18 h posttransfection. (B–F) Electron microscopic images of 293 T cells 48 h after transfection with pEGFP-C3 (B), pEGFP-C3–σ1 (C–D), or pEGFP-C3–μ1 (E–F) identified apoptotic cells with early chromatin condensation (C, E) and late karyorrhexis (D, F). Bar  = 2 μm (E) or 5 μm (B, C, D, F).

We identified the viral factor(s) involved in apoptosis in the widely used 293 T cell system. Genetic studies using T1L × T3D reassortant reoviruses identified the μ1-encoding M2 gene segment as the primary determinant of strain-specific differences in reovirus-induced apoptosis [25]. The ectopic expression of full-length μ1 indicated that the μ1 protein is essential for apoptosis [17], [18], [26]. As well as the μ1 protein, the σ1 protein is also considered to be a determinant of the capacity of reovirus strains to induce apoptosis [27], [28]. However, no similar ectopic study of the σ1 protein has been reported. Therefore, we tested the apoptotic potential of both σ1 and μ1 in transfected cells. The full-length genes encoding BYD1 σ1 and μ1 were cloned into the expression vector pEGFP-C3 fused to the C-terminal of the green fluorescent protein (GFP) sequence. 293 T cells were transfected with vectors expressing GFP alone or the GFP-tagged proteins, and were examined with phase-contrast and fluorescence microscopy. Notably, compared with the control cells transfected with empty vector, 293 T cells transfected with plasmid expressing either GFP–σ1 or GFP–μ1 showed clear cytopathic effects, with characteristic cell shrinkage and partial detachment, suggesting apoptosis-inducing roles for GFP–σ1 and GFP–μ1 (Figure 2). The apoptotic transfected cells were quantified by flow cytometry. Significant proportions of cells expressing GFP–σ1 or GFP–μ1 were apoptotic (Figure 3A). Further electron microscopic analysis at 48 h posttransfection confirmed the presence of ultrastructural changes characteristic of apoptosis in the cells transfected with these two viral genes (Figure 3C–F). Early apoptotic cells with chromatin condensation and late apoptotic cells with ruptured nuclei were observed in the cells expressing GFP–σ1 or GFP–μ1. From these data, we conclude that both the σ1 and μ1 proteins of BYD1 induce apoptosis.

### Multi-organ lesions in suckling mice infected with SARS-MRV

**Figure 4:**
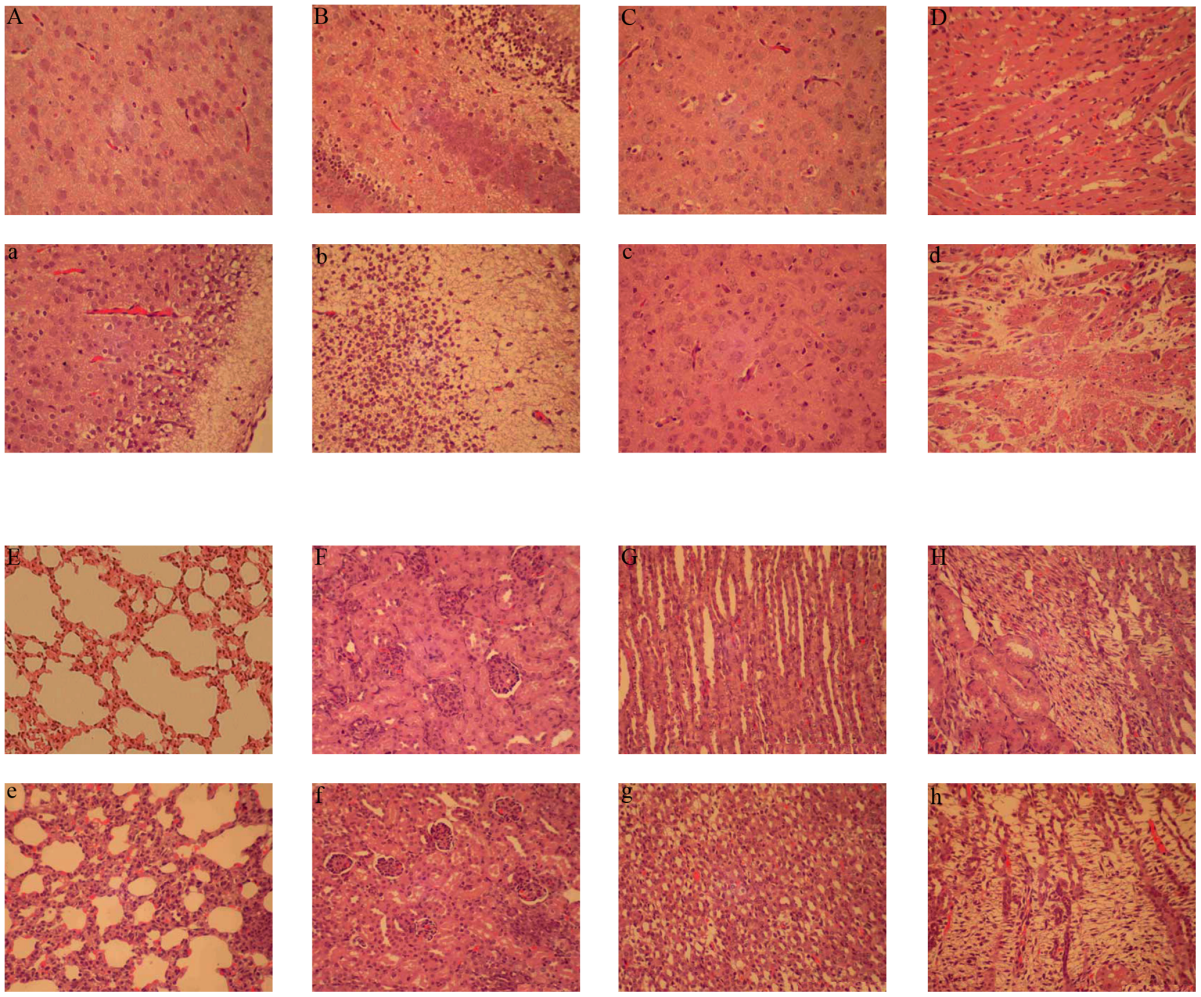
Histopathological evaluation of multiple tissues from mock-infected (A–H) and BYD1-infected (a–h) suckling mice. Micrographs show representative examples of H&E-stained tissues evaluated eight days postinfection at ×200 magnification. The tissue types are pallium (a), hippocampus (b), thalamus (c), cardiac muscle (d), lung (e), renal cortex (f), distal convoluted tubule (g), and nephridial tissue (h). BYD1 infection caused pathological injury to the central nervous tissues (a, b), myocarditis (d), and pneumonia (e) in suckling mice.

Intracranial inoculation of suckling mice with reovirus T3D causes injury to a variety of organs, including the central nervous system (CNS), heart, and liver, which is associated with extensive apoptosis at the sites of viral replication [29]–[31]. To determine whether SARS-MRV infection in suckling mice causes similar diseases, we inoculated suckling mice intracranially with BYD1 or PBS (mock) and monitored the mice for signs of sickness. The mice infected with BYD1 showed symptoms of neurological disease, including heavy breathing, lethargy, and unresponsiveness to external stimulation, at one week postinfection. The mice were then euthanized, and their organs were examined with H&E staining on day 8 following intracranial viral inoculation (Figure 4). A histopathological analysis revealed that the mice infected with BYD1 had signs of central nerve damage, myocarditis, and pneumonia, but no or minor symptoms in the thalamus, renal cortex, distal convoluted tubule, or nephridial tissues. These results suggest a preference of BYD1 for the pallium and hippocampus, and also support the use of suckling mice as a small animal model in which to study the pathogenesis of BYD1. The association between apoptosis and reovirus pathogenesis has been well established in a murine model [32]. Thus, our finding that σ1 and μ1 are inducers of apoptosis suggests that the apoptosis induced by these two proteins contributes to the multi-organ lesions in suckling mice.

## Discussion

Various viruses, such as herpes simplex virus, human immunodeficiency virus, influenza virus, and Hantavirus, can induce apoptosis *in vitro*, which is characterized by ultrastructural changes in the infected cells [33]–[36]. Thus, apoptosis has been accorded an important role in the pathogenesis of some viral infectious diseases. SARS-CoV is accepted as the primary causative agent of SARS by the scientific community. The apoptosis of the host cells caused by SARS-CoV has also been suggested to play a pathogenic role [37], [38]. Although the SARS research community has focused on SARS-CoV, we were interested in investigating the pathogenesis of the novel reassortant SARS-MRV. Reovirus is not recognized as an important human pathogen, but significant human diseases have been attributed to reovirus infection (reviewed in [39]). SARS-MRV has been isolated many times from the lung tissues and throat swabs of SARS patients and anti-SARS-MRV antibodies occur in a proportion of SARS patients, which highlights the importance of SARS-MRV in the SARS epidemic [19]. Our finding that SARS-MRV induces apoptosis in Hep-2 cells supports the possible pathogenic role of apoptosis and is consistent with the well-established fact that reovirus induces apoptosis.

*In vitro* and *in vivo* reovirus infections *are* well characterized systems for studying apoptotic pathogenesis. Previous genetic studies have shown that the μ1-encoding M2 gene is linked to strain-specific differences in the apoptosis-inducing capacity of reovirus (reviewed in [14]). Wisniewski *et al.* [18] found that the ectopic expression of reovirus μ1 mimics reovirus-induced apoptosis in terms of the activation of initiator caspases 8 and 9 and the release of cytochrome *c* and smac/DIABLO from the mitochondrial intermembrane space. However, the same group also found that the ectopic expression of μ1 derived from the relatively nonapoptogenic T1L and apoptogenic T3D reovirus strains induced similar levels of apoptosis. They also showed that the coexpression of σ3 with μ1 abrogated the capacity of μ1 to induce apoptosis [17]. Several possible explanations were proposed for this discrepancy [18]. In addition to the M2 gene, the S1 gene encoding attachment protein σ1 was also recognized in early studies to be a primary determinant of apoptotic efficiency [27], [28], [40]. The σ1 protein is essential for viral attachment and thus for infectivity [41], [42], although the σ1 protein of SARS-MRV varies markedly compared to the σ1 proteins of other reported strains [21]. Our finding that ectopically expressed μ1 and σ1 cause cytopathogenic apoptosis in 293 T cells confirms the previously known function of μ1, and presents a previously unexpected role for cytosolic σ1 as an inducer of apoptosis. We conclude that σ1-induced apoptosis is probably relevant to the pathogenesis of SARS-MRV infection. Further study of the σ1-associated apoptosis pathway should provide novel insight into reovirus-induced apoptosis.

While the contribution of SARS-MRV to SARS is yet to be fully explored, our previous studies in guinea-pig and nonhuman primate model systems supports a pathogenic role for SARS-MRV in SARS. Similarly, our results in the suckling mouse intracranial infection model show that the viral virulence of SARS-MRV is directed against the CNS, which is consistent with the CNS-directed virulence of other reovirus strains. It should be noted that all our four SARS-MRV strains were isolated from severely affected SARS patients; some SARS patients also experience central nervous symptoms during the course of their illness and that SARS-CoV was isolated from a brain tissue specimen obtained from a SARS patient [43]. Whether SARS-MRV is involved in damaging the CNS of SARS patients has never been examined, but is suspected based on our current understanding of SARS-MRV. The lack of broader clinical investigations on SARS-MRV during the 2003 SARS pandemic hindered us to define a co-pathogenic or synergistic role of SARS-MRV and SARS-CoV. Future studies of the impact of co-infection by the two viruses in a nonhuman primate model will likely further our understanding of the pathogenesis of SARS-MRV.
